# Synergistic Effects of Subsurface Drainage and Root-Zone Oxygenation on Wheat Yield and Ion Homeostasis in Saline Soils with Shallow Groundwater

**DOI:** 10.3390/plants15081170

**Published:** 2026-04-10

**Authors:** Qi Xu, Wenda Du, Changkun Ma, Quanjiu Wang

**Affiliations:** 1 State Key Laboratory of Eco-Hydraulics in Northwest Arid Region, Xi’an University of Technology, Xi’an 710048, China; qi_xu0212@163.com (Q.X.); 15130658270@163.com (W.D.); 2 Institute of Arid Region Ecological Water Conservancy, Xi’an University of Technology, Xi’an 710048, China

**Keywords:** gas-phase continuity, rhizosphere hypoxia, potassium-sodium homeostasis, saline soil reclamation, wheat production, waterlogging stress

## Abstract

Shallow groundwater in saline soils creates a self-reinforcing cycle where waterlogging-induced root hypoxia impairs the ATP-dependent sodium exclusion mechanisms that plants rely on for salt tolerance. We conducted a two-year field experiment to test whether subsurface drainage must precede root-zone aeration for oxygen delivery to be effective. The experimental site was located in Heyang County, Weinan City, on the Guanzhong Plain of Shaanxi Province, north-central China—a major alluvial agricultural region representative of shallow-groundwater-induced salinization. The site had saturated paste electrical conductivity of 6.0 dS m^−1^ and groundwater depth fluctuating between 0.5 and 1.4 m. A randomized complete block design with 2 × 2 factorial arrangement compared four treatments: control (CK), subsurface drainage only (SD), root-zone aeration only (RA), and both interventions combined (SD + RA). Drainage increased air-filled porosity from 5.8% to 13.5%, crossing the 10.2% threshold (95% CI: 9.1–11.3%) where gas-phase continuity emerges according to segmented regression analysis. Without drainage, aeration achieved only 4.2 mg L^−1^ dissolved oxygen with high spatial variability (CV 12.5%), while the combined treatment reached 6.8 mg L^−1^ (CV 6.8%). Root ATP content increased by 89% in SD + RA compared to control, accompanied by 56% lower root Na^+^ and 185% higher K^+^/Na^+^ ratio. These physiological changes correlated with 31% higher grain yield (7580 vs. 5798 kg ha^−1^). The synergy index of 1.40 (95% CI: 1.28–1.52) indicated that combined effects exceeded the sum of individual treatments by 40%. Methane emissions declined by 62%, and the system achieved a 2.9-year payback period with a benefit–cost ratio of 4.08. These results establish drainage as a physical prerequisite for effective oxygenation, providing a mechanistic explanation for the variable performance of aeration systems reported in previous studies.

## 1. Introduction

Soil salinization now affects approximately 1.1 billion hectares globally, with an additional 2 million hectares degraded each year [1]. The problem intensifies where shallow groundwater persists—across China’s Huang-Huai-Hai Plain, the Nile Delta, and much of the Indo-Gangetic Basin—because capillary rise continuously replenishes root-zone salts even as saturated conditions compromise root function [2].

The interaction between salinity and waterlogging deserves closer attention than it typically receives. Salt tolerance is energetically expensive. Plants must drive plasma membrane Na^+^/H^+^ antiporters and vacuolar H^+^-ATPases to maintain cytoplasmic ion concentrations within tolerable ranges, processes that depend on adequate ATP supply [3,4]. Under aerobic conditions, respiration yields 30–32 ATP molecules per glucose. When shallow groundwater saturates the root zone, however, gas diffusion rates drop by approximately four orders of magnitude, forcing roots toward fermentative metabolism that produces only 2 ATP per glucose [5,6]. This fifteen-fold reduction in energy availability undermines active Na^+^ exclusion, creating what we might characterize as a positive feedback loop: waterlogging leads to hypoxia, hypoxia reduces ATP production, and reduced ATP impairs the ion regulation needed to tolerate salinity [7,8]. Under combined stress, the effective salt tolerance of many crops drops from 6 to 8 dS m^−1^ to 3–4 dS m^−1^ [4].

Subsurface drainage provides one intervention point in this cycle. By lowering water tables, drainage facilitates salt leaching and typically increases yields by 15–25% in affected areas [9,10]. Drainage alone may not fully restore adequate aeration, though, particularly during intervals between irrigations when capillary water still occupies much of the pore space [11]. Root-zone oxygenation—delivering compressed air directly into the rhizosphere—targets hypoxia more directly and has shown promise in both vegetable and broad-acre systems, with reported yield increases of 15–30% under favorable conditions [12,13].

What has received less attention is the physical requirement for oxygenation to succeed. Gas transport in porous media follows percolation theory: below a critical air-filled porosity (typically 8–12% depending on texture), the gas phase consists of isolated pockets that cannot support bulk diffusion. Above this threshold, a continuous network forms through which injected air can spread [14,15]. When air-filled porosity remains below threshold, injected air tends to escape via preferential flow paths and may never reach the bulk root zone [16]. This physical constraint could explain why oxygenation studies report such variable outcomes, yet no field experiment has directly tested the hypothesis.

We designed a two-year factorial experiment to address this gap. Our working hypotheses were: (H1) subsurface drainage restores air-filled porosity above the percolation threshold; (H2) only after exceeding this threshold does oxygenation work primarily through diffusion rather than preferential flow, producing synergistic rather than merely additive effects; and (H3) the resulting improvement in oxygen availability restores ATP-dependent ion exclusion capacity. To test these hypotheses, the specific objectives of this study were: (1) to quantify the air-filled porosity percolation threshold in a shallow-groundwater saline soil and determine whether subsurface drainage reliably restores porosity above this threshold; (2) to evaluate whether root-zone oxygenation achieves greater dissolved oxygen delivery and spatial uniformity when applied in combination with drainage versus in isolation; (3) to characterize the physiological pathway linking improved soil aeration to root energy status, ion homeostasis, photosynthetic performance, and grain yield; and (4) to assess the agronomic, environmental, and economic outcomes of the integrated drainage-oxygenation system under field conditions.

## 2. Materials and Methods

### 2.1. Site Description

**Figure 1:**
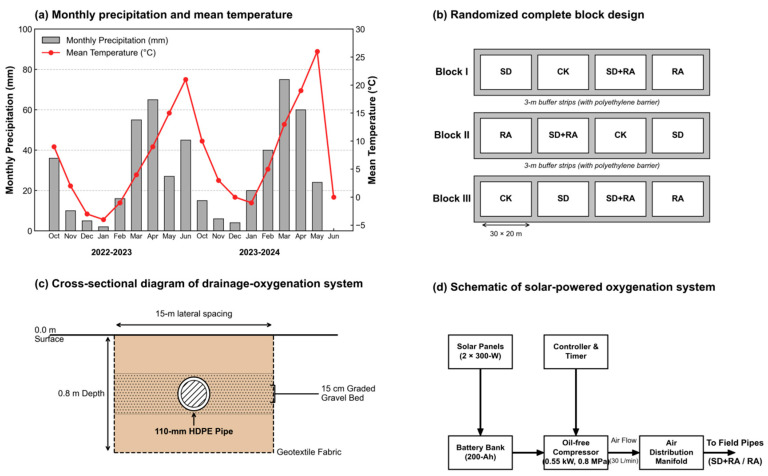
Experimental site characteristics and system configuration. (**a**) Monthly precipitation and mean temperature during growing seasons. (**b**) Randomized complete block design. (**c**) Cross-sectional diagram of the drainage-oxygenation system. (**d**) Schematic of solar-powered oxygenation system.

The experiment was conducted at the Agricultural Experimental Station in Heyang County, Weinan City, China (35°14′40″ N, 110°9′24″ E; 365 m elevation) during winter wheat seasons 2022–2023 and 2023–2024. Heyang County is situated on the eastern Guanzhong Plain—a major alluvial agricultural region formed by the Wei River Basin in north-central China—and is among the areas most seriously affected by shallow-groundwater-induced soil salinization in the region, making it a highly representative experimental site for this study. The climate is warm-temperate continental monsoon, with a mean annual temperature of 11.5 °C, precipitation 528 mm (concentrated July–September), and pan evaporation 1200 mm. Growing-season rainfall totaled 266 mm in Year 1 and 289 mm in Year 2, both below long-term averages and representative of the region’s semi-arid character (Figure 1a).

Soil texture is sandy loam (58% sand, 29% silt, 13% clay by USDA classification). Table 1 summarizes baseline properties. Root-zone EC_1:5_ averaged 1.20 dS m^−1^, which we converted to saturated-paste ECe using a site-specific calibration factor of 5.0 (±0.35 SE) derived from 15 paired measurements following USDA Handbook 60 protocols [17]. The resulting ECe of 6.0 dS m^−1^ classifies the site as moderately saline. Applying the bounds of the conversion factor uncertainty (5.0 ± 0.35 SE) to the measured EC_1:5_ of 1.20 dS m^−1^ yields ECe estimates ranging from 5.58 to 6.42 dS m^−1^, both of which fall within the USDA moderately saline classification range of 4.0–8.0 dS m^−1^, confirming that the site classification is robust to the uncertainty inherent in the conversion factor. Groundwater depth fluctuated between 0.5 m and 1.4 m over the annual cycle, reaching shallowest levels during spring irrigation when wheat is most sensitive to stress.

Initial dissolved oxygen in soil solution was 2.6 mg L^−1^, and redox potential was +148 mV, values consistent with mildly reducing conditions typical of shallow groundwater environments [18].

### 2.2. Experimental Design

Four treatment groups were established: CK (control, no intervention), SD (subsurface drainage only), RA (root-zone aeration only), and SD + RA (drainage combined with aeration), as detailed below. We employed a randomized complete block design with a 2 × 2 factorial structure (Figure 1b). The two factors were subsurface drainage (present or absent) and root-zone aeration (present or absent), yielding four treatments: CK (control, neither intervention), SD (drainage only), RA (aeration only), and SD + RA (both combined). Each treatment was replicated three times across three spatial blocks, giving 12 plots total. Individual plots measured 30 × 20 m.

Treatment positions within each block were randomly assigned using computer-generated sequences (R v4.2.0) before any installation began. To verify baseline homogeneity, we sampled five points per plot (60 total) in August 2022. Coefficients of variation were 8.5% for electrical conductivity, 6.8% for organic matter, 7.2% for groundwater depth, and 4.2% for bulk density. One-way ANOVA detected no pre-treatment differences among plots (all *p* > 0.25). Moran’s I test for spatial autocorrelation was not significant (I = 0.08, *p* = 0.32), confirming the absence of systematic spatial gradients that might confound treatment comparisons.

Plots were separated by 3 m buffer strips with polyethylene film barriers extending to 1.8 m depth to prevent lateral water and salt movement between plots.

One design feature is central to our interpretation: the RA treatment used pipes physically identical to those in SD treatments, but with their outlets permanently sealed rather than connected to drainage wells. Consequently, RA pipes filled with soil water during high-groundwater periods and could not drain. Injected air had to traverse saturated soil—mimicking real-world conditions where farmers attempt oxygenation without prior drainage infrastructure. The SD and SD + RA pipes connected to collection wells and drained freely, ensuring air-filled pipes when oxygenation occurred. Both configurations involved identical excavation and backfill, so any soil disturbance effects were equivalent; the sole difference was hydraulic versus pneumatic function.

After harvest, we conducted ANCOVA including plot coordinates (northing, easting) as covariates to check for any residual spatial effects. Neither coordinate significantly affected yield or physiological variables (all *p* > 0.35), and treatment effects remained essentially unchanged—confirming that observed differences reflect treatments rather than uncontrolled spatial gradients.

Post hoc power analysis (G*Power 3.1) based on observed effect sizes indicated statistical power of 0.87 for main effects and 0.78 for the interaction term at α = 0.05, both exceeding conventional thresholds for adequate power.

### 2.3. Drainage and Oxygenation System

Drainage pipes were 110 mm corrugated HDPE with 20 cm^2^ m^−1^ perforation area, installed at 0.8 m depth with 15 m lateral spacing and 0.3% grade. Pipes were enveloped in 200 g m^−2^ geotextile fabric and bedded in 15 cm of graded gravel (5–20 mm) (Figure 1c).

The 0.8 m installation depth was selected based on several considerations. Preliminary root excavations showed that 85% of wheat root mass at this site occurred within 0–60 cm depth, consistent with documented root depth distributions for temperate wheat under comparable soil conditions [19]. We aimed to maintain groundwater below 70 cm during critical growth stages while avoiding root intrusion into pipes. The 15 m lateral spacing was calculated using the Hooghoudt equation with measured saturated hydraulic conductivity (1.2 × 10^−5^ m s^−1^) to achieve the target drawdown within 48 h of irrigation [20].

The oxygenation system included two 300 W solar panels, a 200-Ah battery bank, an oil-free compressor (0.55 kW, 0.8 MPa maximum capacity), and an air distribution manifold (Figure 1d). Oxygenation events were initiated 48 h after each irrigation—the time point at which drained plots had recovered air-filled porosity above the percolation threshold. Each event ran 4 h (09:00–13:00) at 30 L min^−1^, delivering approximately 80 m^3^ ha^−1^. There were roughly 18 oxygenation events per growing season. SF_6_ tracer tests confirmed reasonably uniform air distribution (spatial CV = 6.8%, Christiansen uniformity coefficient = 92.5%).

### 2.4. Crop Management

Table 2 details field operations. Winter wheat cv. ‘Jimai 22’, a widely planted semi-dwarf cultivar adapted to this region, was obtained from the Crop Research Institute of Shaanxi Academy of Agricultural Sciences (Xi’an, China) and sown in early October at a 225 kg ha^−1^ seed rate. Nitrogen was applied at 159 kg N ha^−1^ total as urea, split 40:60 between basal application and jointing topdressing—a schedule designed to match supply with crop demand while limiting leaching risk [21]. Phosphorus (90 kg P_2_O_5_ ha^−1^) and potassium (75 kg K_2_O ha^−1^) were applied basally at sowing.

Irrigation water came from deep wells (80 m depth; EC = 0.65 dS m^−1^; SAR = 2.1)—water classified as excellent quality under FAO guidelines. We applied 45 mm at each of four phenological stages (overwintering, jointing, anthesis, grain-filling) for 180 mm total seasonal application. The low salinity of irrigation water ensured that soil salinity dynamics reflected treatment effects rather than irrigation inputs.

### 2.5. Measurements

Groundwater and soil physical properties. Automated pressure transducer loggers (HOBO U20L-04; ±0.1% accuracy) recorded hourly water-table depth in observation wells at plot centers. Air-filled porosity was measured on undisturbed 100-cm^3^ cores collected at 10–20 cm depth at 24, 48, 72, and 96 h after irrigation (three cores per plot per sampling time). Total porosity was calculated from particle and bulk densities; air-filled porosity was computed as total porosity minus volumetric water content.

Gas diffusivity. Relative gas diffusivity (Dp/D0) was measured by the one-chamber method on undisturbed cores equilibrated to field moisture conditions [22]. Oxygen concentration decay was logged at 1 min intervals; Dp was calculated from exponential decay kinetics. We fitted both Millington-Quirk and Deepagoda models to the εa–Dp/D0 relationship for comparison.

Dissolved oxygen. Ceramic-cup microsamplers (Rhizon SMS; 0.15-μm pore size) were installed at 15 cm depth at three distances from oxygenation pipes (0, 3.75, 7.5 m radially), with three replicates per distance, giving nine sampling points per plot. A portable optical meter (YSI ProODO; ±0.2 mg L^−1^ precision) recorded dissolved oxygen at six time points during each irrigation-oxygenation cycle: before irrigation (T0), 2 h after irrigation (T1), 48 h after irrigation just before oxygenation (T2), 2 h after oxygenation (T3), 24 h post-oxygenation (T4), and 72 h post-oxygenation (T5). This spatial array characterized both mean dissolved oxygen levels and their variability—a diagnostic of the dominant transport mechanism.

Root physiology. At jointing, anthesis, and grain-filling stages, five random plants per plot were sampled. Roots from 0 to 40 cm depth were excavated using a systematic soil monolith approach: a steel frame (20 × 20 cm cross-section) was inserted to the target depth alongside each sampled plant, and the enclosed soil block was extracted intact to minimize root severance and loss. The soil monolith was then carefully broken apart by hand and immersed in water to allow roots to float free from the soil matrix. Root material was subsequently recovered by washing over 0.5 mm sieves in three successive rinses until the rinse water ran clear, ensuring that fine roots adhering to soil aggregates were fully retrieved. The completeness of root recovery was verified by visually inspecting the residual soil material remaining on the sieve after each wash. Flash-freezing in liquid nitrogen was performed within 3 min of final collection to preserve ATP. Samples were stored at −80 °C and analyzed within 72 h. ATP was quantified by luciferase–luciferin bioluminescence using an ATP assay kit (Beyotime Biotechnology, Shanghai, China) and measured on a GloMax 20/20 luminometer (Promega Corporation, Madison, WI, USA). We ran fresh standard curves (R^2^ > 0.99) with each analytical batch; intra-assay CV averaged 4.2% and inter-assay CV was 7.8%. Separate root subsamples were oven-dried (75 °C, 48 h), acid-digested (HNO_3_-HClO_4_ mixture), and analyzed for K^+^ and Na^+^ by flame photometry (precision ±2.5%).

Photosynthesis. Flag-leaf gas exchange was measured at anthesis using a portable photosynthesis system (LI-6400XT; LI-COR Biosciences, Lincoln, NE, USA) under standardized conditions (1500 μmol m^−2^ s^−1^ PAR, 400 μmol mol^−1^ CO_2_, 25 °C leaf temperature). Mesophyll conductance was estimated using the variable-J method [23].

Greenhouse gases. Static chambers (50 × 40 × 60 cm) were sampled at 0, 10, 20, and 30 min intervals; gas samples were analyzed by gas chromatography with FID detection for CH_4_ and ECD detection for N_2_O. Sampling occurred weekly throughout the growing season, with additional measurements within 48 h of irrigation or fertilization events. Flux calculations were rejected if the linear regression R^2^ < 0.90. Global warming potential was calculated using IPCC AR6 characterization factors (CH_4_ × 27.9, N_2_O × 273 for a 100-year timeframe) [24].

Yield and resource use efficiency. Grain yield was determined from three 1-m^2^ quadrats per plot, adjusted to 13% moisture content. Water productivity was calculated as yield divided by actual evapotranspiration estimated by soil water balance [25]. Partial factor productivity of nitrogen was computed as yield divided by total N applied.

Drainage water quality. Samples were collected after each irrigation event and analyzed for NO_3_^−^-N by ion chromatography (precision ±5%).

### 2.6. Economic Analysis

Net present value was calculated over an assumed 15-year system lifetime at 6% discount rate, including straight-line depreciation of the initial capital investment. Sensitivity analysis tested ±30% variation in yield benefit, ±50% variation in installation costs, and discount rates ranging from 4% to 10%.

### 2.7. Statistical Analysis

Prior to statistical analysis, data were examined for conformity with the assumptions underlying parametric testing. Normality of residuals was assessed using the Shapiro–Wilk test (α = 0.05), and homogeneity of variance was evaluated using Levene’s test (α = 0.05). All key response variables—including grain yield, root ATP content, dissolved oxygen, and K^+^/Na^+^ ratio—satisfied both assumptions (Shapiro–Wilk *p* > 0.05; Levene’s test *p* > 0.05), confirming that parametric analysis was appropriate. Two-way ANOVA was then performed to test the main effects of drainage and aeration and their interaction, with experimental block included as a random effect. All statistical analyses were conducted in R version 4.2.0. We report Cohen’s d effect sizes for key comparisons. The synergy index:

(1) $$\mathrm{SI}=\frac{Y_{\mathrm{SD}+\mathrm{RA}}-Y_{\mathrm{CK}}}{\left(Y_{\mathrm{SD}}-Y_{\mathrm{CK}}\right)+\left(Y_{\mathrm{RA}}-Y_{\mathrm{CK}}\right)}$$

where *Y* denotes the two-year mean grain yield (kg ha^−1^) for each respective treatment. The numerator quantifies the observed yield increment of the combined treatment (SD + RA) relative to the untreated control (CK), while the denominator represents the expected additive increment—that is, the arithmetic sum of the yield gains produced independently by subsurface drainage alone (SD) and root-zone aeration alone (RA), each measured against CK. where *SI* > 1 indicates super-additive synergy, *SI* = 1 indicates additive effect, and *SI* < 1 indicates antagonism. *SI* was calculated with 95% confidence intervals derived from 10,000 bootstrap iterations. Segmented regression identified the air-filled porosity threshold using the segmented package in R; Davies’ test compared segmented versus simple linear model fits.

The segmented model was selected over a simple linear regression because Davies’ test confirmed a statistically significant improvement in fit (*p* < 0.001), indicating that a single-slope model was insufficient to describe the εa–dissolved oxygen relationship across the full observed range.

To validate the threshold estimate, we randomly split the pooled εa–dissolved oxygen dataset (*n* = 216) into calibration (70%) and validation (30%) subsets. The threshold identified from the calibration subset (10.3%) fell within the 95% confidence interval of the full-dataset estimate (9.1–11.3%), and applying this threshold to the validation subset produced similar breakpoint behavior (validation RMSE = 0.42 mg L^−1^).

Partial least squares path modeling explored associations among variables. The PLS-PM was constructed using plot-level observations pooled across both growing seasons (*n* = 24; 12 plots × 2 years). Root physiological variables were averaged across the three measurement stages (jointing, anthesis, and grain-filling), and photosynthetic variables were represented by anthesis-stage values. This sample size satisfies the ten-times rule for PLS-PM, which requires a minimum of ten observations per maximum number of structural paths directed at any endogenous construct. The significance of all path coefficients was assessed by bootstrap resampling (999 iterations). We emphasize that path coefficients reflect correlational relationships and should not be interpreted as proof of causation without supporting mechanistic evidence.

## 3. Results

### 3.1. Groundwater Dynamics and Soil Physical Properties

**Figure 2:**
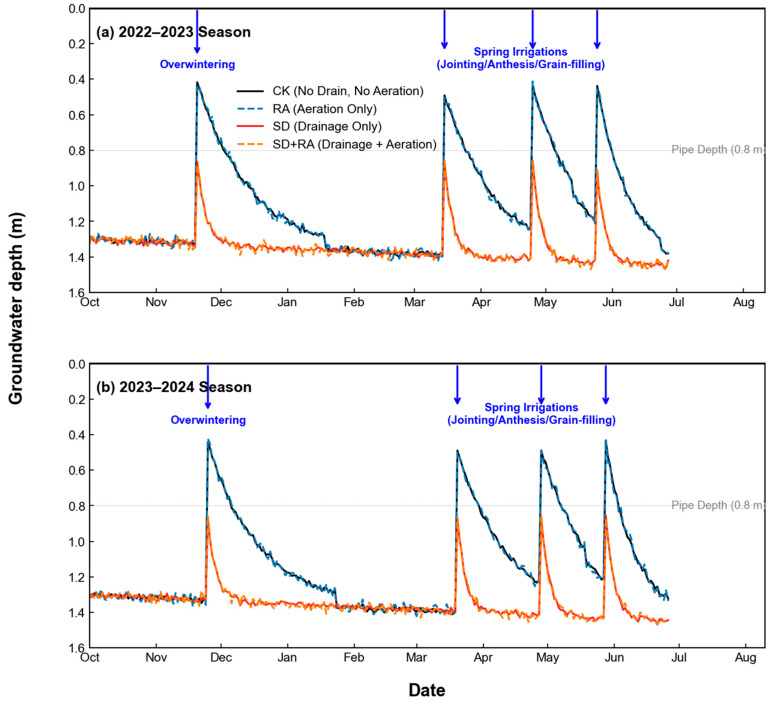
Groundwater depth dynamics across two growing seasons. Horizontal dashed line indicates 0.7 m critical depth threshold.

Subsurface drainage lowered the average water table position by 0.28 m compared to non-drained treatments (Figure 2). In CK and RA plots, groundwater remained shallower than 0.7 m for 38–45 days each season, predominantly during spring irrigation when wheat enters reproductive stages. SD and SD + RA plots maintained groundwater between 0.85 m and 1.35 m depth throughout both growing seasons.

Air-filled porosity at 48 h post-irrigation—the timing when oxygenation was applied—reached 12.3–13.5% in drained treatments compared to 5.8–6.2% in non-drained treatments (Table 3). This timing is significant: at 48 h, drained plots had reestablished gas connectivity while irrigation water still occupied much of the pore space in non-drained plots.

**Figure 3:**
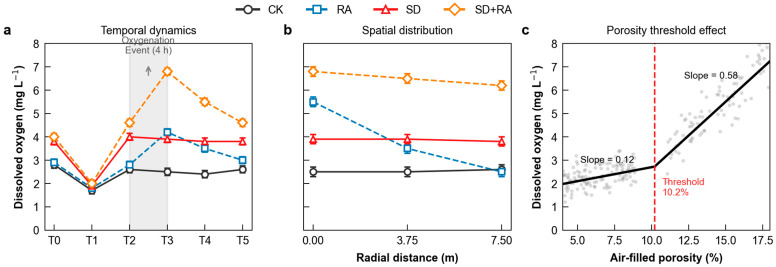
Dissolved oxygen dynamics and percolation threshold identification. (**a**) Temporal dynamics through irrigation-oxygenation cycles. (**b**) Spatial distribution at T3. (**c**) Segmented regression of dissolved oxygen against air-filled porosity (εa) across pooled observations (*n* = 216).

Segmented regression applied to the pooled εa–dissolved oxygen dataset (*n* = 216 observations) identified a breakpoint at air-filled porosity of 10.2% (95% CI: 9.1–11.3%; Davies’ test for improved fit *p* < 0.001; Figure 3c). Below this threshold, dissolved oxygen showed a weak response to air-filled porosity (slope = 0.12 mg L^−1^ per percentage point). Above the threshold, dissolved oxygen increased steeply (slope = 0.58 mg L^−1^ per percentage point). This nearly five-fold difference in slopes marks the transition from disconnected to connected gas phase that percolation theory predicts. The identified threshold of 10.2% falls within the 8–12% range predicted by percolation theory for sandy loam soils [14] and is consistent with the approximately 10% value reported by Moldrup et al. [15] for soils of comparable texture and bulk density (1.40–1.55 g cm^−3^). The convergence between the segmented regression result, the cross-validation breakpoint (10.3%), and the gas diffusivity inflection point provides three independent lines of evidence that the identified value reflects a genuine physical transition rather than a statistical artifact.

Gas diffusivity measurements supported these findings (Table 4). Relative gas diffusivity in non-drained plots (0.008–0.010) fell well below the 0.02 threshold commonly cited as necessary for adequate root respiration [26]. Drained plots achieved Dp/D0 of 0.042–0.048, roughly doubling this benchmark. The Deepagoda model provided a better fit to our data (R^2^ = 0.94) than the Millington-Quirk model (R^2^ = 0.87), and the slope of Dp/D0 versus εa increased 5.7-fold above the 10% threshold—further evidence of the percolation transition.

Soil salinity showed differential responses to treatments over the two-year period (Table 5). Root-zone EC_1:5_ in SD + RA decreased from 1.19 to 0.93 dS m^−1^, representing a 21.8% reduction—the largest among all treatments. SD alone achieved 16.9% reduction (from 1.18 to 0.98 dS m^−1^), while CK and RA showed minimal changes (−2.5% and −3.3%, respectively). These patterns indicate that drainage facilitates salt leaching, with combined drainage-oxygenation providing the greatest desalinization benefit.

### 3.2. Dissolved Oxygen Dynamics and Spatial Patterns

Dissolved oxygen followed a characteristic temporal pattern after irrigation (Figure 3a). At T1 (2 h post-irrigation), dissolved oxygen dropped to 1.5–2.0 mg L^−1^ across all treatments as irrigation water displaced soil air. By T2 (48 h), partial recovery had occurred: CK reached 2.6, RA 2.8, SD 4.0, and SD + RA 4.6 mg L^−1^. The fact that SD exceeded CK by 54% without any artificial air input demonstrates that drainage alone promotes atmospheric oxygen re-entry by restoring gas connectivity.

At T3 (2 h after oxygenation), SD + RA peaked at 6.8 mg L^−1^—70% above concurrent SD levels (*p* < 0.001; Cohen’s d = 2.6). Despite receiving identical oxygenation equipment and air volumes, RA achieved only 4.2 mg L^−1^, which is 38% lower than SD + RA. This performance gap cannot be attributed to insufficient air supply; rather, it reflects transport limitations in saturated soil.

Spatial patterns provided additional diagnostic information about transport mechanisms (Figure 3b). SD + RA showed relatively uniform dissolved oxygen across the 7.5 m radial transect (CV = 6.8%), consistent with diffusive spread through a connected pore network. RA displayed steeper spatial gradients (CV = 12.5%), with dissolved oxygen highest near injection points and declining with distance—a pattern characteristic of preferential flow through macropores. In RA plots, air bubbles frequently broke the soil surface within minutes of injection initiation; no such surface bubbling occurred in SD + RA treatments.

Elevated dissolved oxygen persisted longer where diffusion-based transport dominated. At 72 h post-oxygenation (T5), SD + RA still maintained 4.6 mg L^−1^ (68% of peak), while RA had declined to 3.0 mg L^−1^. Season-long mean dissolved oxygen levels were: SD + RA 5.6, SD 3.8, RA 3.4, CK 2.6 mg L^−1^.

Redox potential tracked dissolved oxygen closely (r = 0.91; Table 6). CK remained at +148 mV throughout, indicative of mildly reducing conditions. SD + RA reached +205 mV, crossing the approximate +180 mV threshold below which methanogenesis becomes thermodynamically favorable.

### 3.3. Root Energy Status and Ion Homeostasis

**Figure 4:**
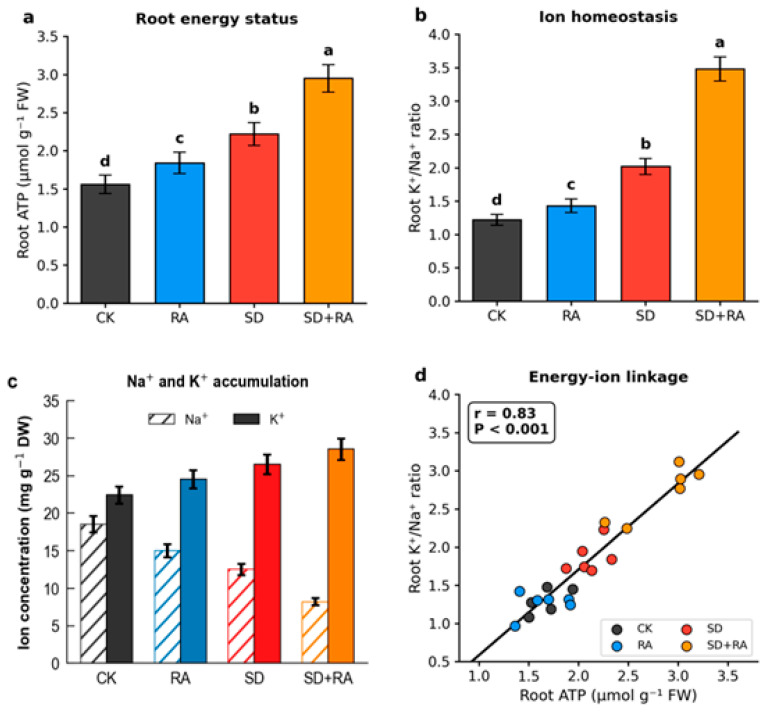
Root physiological responses. (**a**) Root ATP content across growth stages. (**b**) Root ion concentrations at anthesis. (**c**) Root ion concentration. (**d**) Correlation between ATP and K^+^/Na^+^ ratio. Different lowercase letters on the figure columns indicate significant differences between treatments (*p* < 0.05).

Root ATP content showed substantial treatment effects (Figure 4a). SD + RA reached 2.95 μmol g^−1^ fresh weight at anthesis—89% above CK (1.56 μmol g^−1^; *p* < 0.001; Cohen’s d = 2.9), 33% above SD alone (2.22 μmol g^−1^), and 60% above RA alone (1.84 μmol g^−1^). Across all four treatments, ATP content at anthesis increased in the order CK (1.56) < RA (1.84) < SD (2.22) < SD + RA (2.95) μmol g^−1^ FW, with all pairwise differences statistically significant (*p* < 0.05, Tukey’s HSD), confirming a consistent positive relationship between the degree of soil aeration and root energy status. This near-doubling of ATP content is physiologically plausible given the fifteen-fold difference in ATP yield between aerobic and fermentative pathways. This interpretation is grounded in the dissolved oxygen data reported in Section 3.2: peak dissolved oxygen rose from 2.6 mg L^−1^ in CK to 6.8 mg L^−1^ in SD + RA at T3, and the season-long mean increased from 2.6 to 5.6 mg L^−1^, together indicating a sustained shift toward aerobic conditions that is consistent with the observed recovery in root ATP. Our oxygenation raised dissolved oxygen to roughly 75% of air saturation, which should restore much—though not all—of the aerobic capacity.

The RA treatment increased ATP by only 18% relative to CK—less than half the effect of SD alone. Spatially patchy and temporally transient oxygen delivery apparently failed to sustain energy metabolism as effectively as the more uniform, diffusion-mediated oxygen supply in drained soils. The dissolved oxygen spatial data reported in Section 3.2 support this directly: the coefficient of variation across the radial sampling transect was 12.5% in RA plots versus 6.8% in SD + RA plots at T3, indicating that a substantial proportion of the root system in RA plots continued to experience sub-threshold oxygen concentrations even at peak injection. Roots occupying these oxygen-deficient micro-zones would have maintained fermentative metabolism, suppressing the whole-root ATP average. This suggests that spatial uniformity of oxygen delivery—not merely its mean concentration—is a critical determinant of the whole-root ATP response.

Ion concentration data paralleled the ATP findings (Figure 4b,c). Root Na^+^ concentration in SD + RA was 8.2 mg g^−1^ dry weight at anthesis—56% below CK (18.5 mg g^−1^; *p* < 0.001; Cohen’s d = 3.2). Root K^+^ increased by 27% (to 28.5 mg g^−1^), and the K^+^/Na^+^ ratio reached 3.48—185% above CK (1.22) and approaching the optimum of approximately 4.0 reported for salt-tolerant wheat genotypes [27].

ATP content and K^+^/Na^+^ ratio showed a strong positive correlation across treatments and growth stages (r = 0.83, *p* < 0.001; Figure 4d). This correlation remained after controlling for soil electrical conductivity differences in ANCOVA (partial r = 0.82), suggesting an independent effect of oxygenation on ion regulation. The pattern is consistent with ATP-dependent Na^+^ exclusion mechanisms, though we did not directly measure transporter activity or gene expression in this study.

### 3.4. Photosynthetic Performance and Root Development

Flag-leaf net photosynthesis rate in SD + RA averaged 21.2 μmol m^−2^ s^−1^ at anthesis—23% above CK (17.3 μmol m^−2^ s^−1^). Stomatal conductance increased by 40%, yet intercellular CO_2_ concentration decreased by 15% (to 229 μmol mol^−1^). This combination of a higher assimilation rate with lower intercellular CO_2_ indicates relief of mesophyll rather than stomatal limitation. Mesophyll conductance increased by 29%, and net photosynthesis correlated more strongly with mesophyll conductance (r = 0.82) than with stomatal conductance (r = 0.58).

The correlation between the K^+^/Na^+^ ratio and mesophyll conductance (r = 0.76) suggests that ionic balance supports mesophyll function. Elevated cytoplasmic Na^+^ can cause cell shrinkage, impair aquaporin function, and disrupt chloroplast positioning—all of which reduce mesophyll conductance [28]. Restoring ion homeostasis likely contributed to the observed photosynthetic improvements.

Root length density at anthesis was 3.25 cm cm^−3^ in SD + RA—48% above CK (2.19 cm cm^−3^; Table 6). Root dry weight was 1.78 g plant^−1^, representing a 62% increase over the control. By contrast, RA achieved only a 9% increase in root length density, again illustrating limited efficacy without drainage.

### 3.5. Greenhouse Gas Emissions and Environmental Impacts

**Figure 5:**
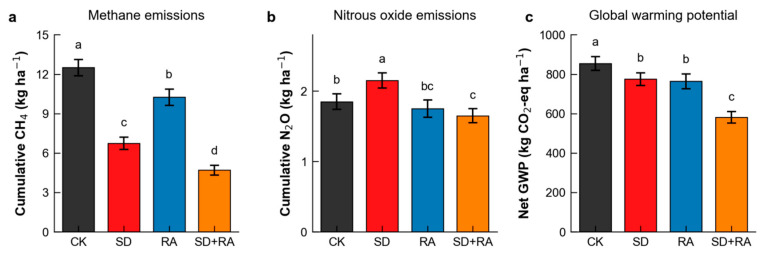
Greenhouse gas emissions and global warming potential. (**a**) Cumulative methane emissions. (**b**) Cumulative nitrous oxide emissions. (**c**) Net global warming potential. Different lowercase letters on the figure columns indicate significant differences between treat-ments (*p* < 0.05).

Cumulative methane emission was lowest in SD + RA at 4.7 kg ha^−1^—62% below CK (12.5 kg ha^−1^; Figure 5a). SD alone reduced methane by 46%; RA reduced it by only 18% (Reduction ratios were calculated as the percentage decrease in cumulative seasonal CH_4_ emission relative to CK: SD, (12.5 − 6.75)/12.5 = 46%; RA, (12.5 − 10.25)/12.5 = 18%; SD + RA, (12.5 − 4.7)/12.5 = 62%). Sustained elevated dissolved oxygen kept redox potential above the threshold for methanogenesis throughout most of the growing season.

Nitrous oxide responses differed somewhat (Figure 5b). SD increased N_2_O emission by 16% (to 2.15 kg ha^−1^), probably through enhanced coupled nitrification-denitrification under fluctuating redox conditions. SD + RA decreased N_2_O by 11% relative to CK (to 1.65 kg ha^−1^), presumably because sustained aeration suppressed denitrification and improved crop nitrogen uptake, reducing substrate availability for denitrifiers.

Net global warming potential in SD + RA was 569 kg CO_2_-eq ha^−1^—32% below CK—with no evidence of pollution swapping (Figure 5c). Greenhouse gas intensity fell from 145 to 75 kg CO_2_-eq per megagram of grain produced.

Drainage water quality remained within regulatory limits throughout both seasons (Table 7). NO_3_^−^-N concentrations averaged 8.9 mg L^−1^, remaining below China’s Class III water quality standard (20 mg L^−1^) and well below the WHO drinking water guideline (50 mg L^−1^). Concentrations peaked at 12–15 mg L^−1^ within 72 h of nitrogen topdressing but declined below 8 mg L^−1^ within two weeks. Seasonal nitrogen loads through drainage (5.0 ± 0.7 kg N ha^−1^) represented roughly 3% of applied nitrogen—comparable to well-managed drainage systems elsewhere.

### 3.6. Yield Response and Synergistic Effects

**Figure 6:**
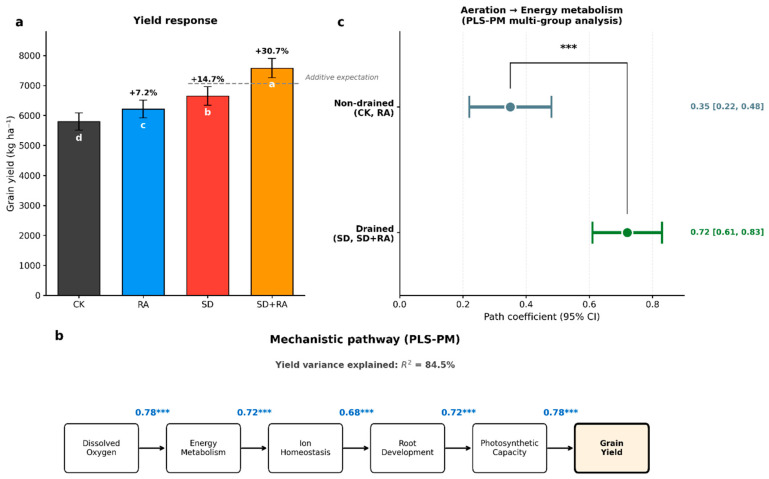
Yield response and mechanistic pathway analysis. (**a**) Two-year mean grain yield by treatment; (**b**) Partial least squares path model (PLS-PM); (**c**) Aeration → energy metabolism path coefficients stratified by drainage status. Different letters indicate significant differences (*p* < 0.05, Tukey’s HSD). Error bars indicate standard deviation (*n* = 6) for panel (**a**) and 95% confidence intervals for panel (**c**). *** denotes a *p*-value of less than 0.001, indicating an extremely high level of statistical significance.

Two-year mean grain yields were: CK 5798, SD 6652 (+14.7%), RA 6215 (+7.2%), and SD + RA 7580 kg ha^−1^ (+30.7%; *p* < 0.001; Cohen’s d = 2.7; Figure 6a). Neither the year main effect nor the year × treatment interaction was statistically significant, indicating consistent treatment responses across seasons despite differences in rainfall distribution.

Yield component analysis (Table 6) showed that SD + RA achieved 465 × 10^4^ spikes ha^−1^ (+10% vs. CK), 37.8 grains per spike (+12%), and 44.5 g thousand-grain weight (+8%). Theoretical yield calculated from these components (465 × 10^4^ × 37.8 × 44.5/1000 = 7823 kg ha^−1^) matched measured yield (7580 kg ha^−1^) within 3.2%—well within the expected range for field harvest losses—confirming internal data consistency.

Water productivity reached 1.74 kg m^−3^ in SD + RA, a 26% improvement over control. Partial factor productivity of nitrogen was 47.7 kg grain per kg N applied (+31% vs. CK).

Two-way ANOVA revealed statistically significant drainage × aeration interactions for dissolved oxygen (F = 25.6, *p* < 0.001), K^+^/Na^+^ ratio (F = 22.4), ATP content (F = 18.5), grain yield (F = 16.8), and methane emission (F = 14.2; Table 8). These interactions confirm that aeration efficacy depends on drainage status.

For yield, drainage alone increased yield by 854 kg ha^−1^ relative to control (SD: 6652 vs. CK: 5798 kg ha^−1^); aeration alone increased yield by 417 kg ha^−1^ (RA: 6215 vs. CK: 5798 kg ha^−1^). The expected additive response would therefore be 1271 kg ha^−1^; the observed combined response was 1782 kg ha^−1^ (SD + RA: 7580 vs. CK: 5798 kg ha^−1^; Table 6; Figure 6a). This gives a synergy index of 1.40 (95% CI: 1.28–1.52; bootstrap test *p* < 0.001), meaning the combined treatment exceeded additive expectations by 40%.

Partial least squares path modeling explained 84.5% of yield variance (Figure 6b). Standardized path coefficients were: dissolved oxygen → energy metabolism (0.78), energy metabolism → ion homeostasis (0.72), ion homeostasis → root development (0.68), root development → photosynthetic capacity (0.72), and photosynthetic capacity → grain yield (0.78). When we stratified the analysis by drainage status, the aeration → energy metabolism coefficient was 0.72 in drained plots versus 0.35 in non-drained plots—quantitatively demonstrating how drainage enables oxygenation effectiveness.

### 3.7. Economic Viability and System Durability

Initial capital investment totaled 8800 CNY ha^−1^ (approximately 1220 USD at current exchange rates; Table 9). Annual operating costs were 987 CNY ha^−1^, including 587 CNY ha^−1^ depreciation over the 15-year expected system lifetime. Net annual benefit was 4092 CNY ha^−1^; simple payback period was 2.2 years; discounted payback was 2.9 years; 15-year net present value was 31,540 CNY ha^−1^; benefit–cost ratio was 4.08. The system remained economically viable under all sensitivity scenarios we tested: with 30% lower yield benefit, BCR = 2.5; with 50% higher installation cost, BCR = 2.7; at 10% discount rate, BCR = 3.2.

Post-experiment excavation of twelve 1 m pipe sections revealed no structural damage, no geotextile tears, and no root intrusion into pipes. Some trace reddish deposits were visible (ochre severity rating 0.8 on a 0–3 scale), affecting less than 5% of the internal pipe surface without blocking perforations. Drainage water iron concentration averaged 1.2 mg L^−1^. Published guidelines indicate that iron concentrations exceeding 1–2 mg L^−1^ in drainage effluent warrant preventive maintenance consideration, while concentrations above 5 mg L^−1^ are associated with operationally significant perforation blockage and measurable reductions in drainage capacity [29,30]. Our observed concentration of 1.2 mg L^−1^ places the system within the low-to-moderate risk category; nonetheless, given that ochre deposition is cumulative and may accelerate under seasonal redox fluctuations, we recommend annual pipe flushing as a low-cost precautionary measure consistent with standard practice for installations operating above 1 mg L^−1^ [29].

## 4. Discussion

### 4.1. Physical Prerequisite for Effective Oxygenation

Three independent lines of evidence support our hypothesis that drainage must restore air-filled porosity above the percolation threshold before oxygenation can function primarily through diffusion.

First, direct gas diffusivity measurements showed that drained plots achieved Dp/D0 of 0.042–0.048—well above the 0.02 threshold generally considered necessary for adequate root respiration—while non-drained plots remained at 0.008–0.010, which is insufficient to meet root oxygen demand. The 5.7-fold increase in the slope of Dp/D0 versus εa above 10% air-filled porosity provided quantitative confirmation of the percolation transition.

Second, spatial dissolved oxygen patterns revealed distinctly different transport regimes. The 1.8-fold difference in coefficient of variation (12.5% in RA versus 6.8% in SD + RA) reflects the contrast between preferential flow through isolated macropores and more uniform diffusion through an interconnected pore network. Visual observations of surface bubbling in RA plots confirmed rapid vertical air losses through preferential pathways.

Third, yield responses quantified the practical consequences of these physical differences. Despite receiving identical air volumes with identical equipment, RA achieved only 7.2% yield increase compared to 30.7% for SD + RA—a more than four-fold disparity attributable primarily to transport limitations.

The threshold we identified (10.2%; 95% CI: 9.1–11.3%) falls within the 8–12% range predicted by percolation theory for sandy loam textures [14], and is in close agreement with the ~10% value reported by Moldrup et al. [15] for soils with similar particle-size distribution and bulk density under field-moist conditions. The 5.7-fold increase in the slope of Dp/D0 versus εa above 10% air-filled porosity (Table 4) provides additional quantitative confirmation, as this inflection in gas diffusivity is the physical signature of the percolation transition in porous media. We should note, however, that this threshold value is texture-specific. Clay soils with smaller pores and greater tortuosity will likely require 12–14% air-filled porosity to achieve equivalent gas connectivity. In the present study, the threshold applies specifically to a sandy loam with 58% sand, bulk density 1.42–1.52 g cm^−3^, and moderate organic matter content (7.5–11.5 g kg^−1^); soils with markedly different texture, structure, or pore-size distribution should be treated as requiring independent threshold determination. Site-specific threshold determination through Dp/D0–εa relationships should ideally precede system design in other regions.

A mechanistic clarification may be helpful here. Percolation theory describes gas-phase transport through soil pores, yet our primary outcome of interest is dissolved oxygen in soil solution. The connection operates through mass transfer at air-water interfaces. When air-filled porosity exceeds the percolation threshold, the continuous gas network increases interfacial area and shortens diffusion path lengths, allowing atmospheric oxygen to dissolve into soil water following Henry’s Law. In isolated gas clusters below threshold, biological consumption creates local depletion that cannot be replenished from the atmosphere. Gas connectivity—fundamentally a physical property—thus governs dissolved oxygen availability, which is the biologically relevant outcome.

### 4.2. Energy Metabolism and Ion Regulation

The 89% increase in root ATP content and the 185% improvement in K^+^/Na^+^ ratio, together with their strong correlation (r = 0.83), are consistent with the hypothesis of ATP-dependent ion exclusion. The theoretical basis is well established: SOS1-mediated Na^+^ efflux and plasma membrane H^+^-ATPase activity both require ATP [4,31]. Several controlled-environment studies have shown that hypoxia reduces H^+^-ATPase activity by 40–60%, with corresponding Na^+^ accumulation [6]. Our field data fit this mechanistic framework.

We should be explicit about what we measured and what we did not. We quantified ATP content and ion concentrations in root tissues; we did not directly measure transporter activity, gene expression patterns, or Na^+^ efflux kinetics. The correlation between ATP and ionic homeostasis supports the mechanistic linkage but does not definitively prove it. More complete molecular validation of the proposed energetic mechanism remains a priority for future work. Three lines of investigation, each targeting a distinct node in the causal chain from aeration to ion homeostasis, should be prioritized: First, quantitative RT-PCR and immunoblotting for SOS1 (encoding the plasma-membrane Na^+^/H^+^ antiporter responsible for active Na^+^ efflux from root cortical cells) and HKT1;5 (encoding the high-affinity transporter mediating Na^+^ retrieval from the xylem stream) would directly test whether the observed improvement in Na^+^ exclusion under aerated conditions reflects transcriptional upregulation of these transporters, as opposed to post-translational regulation or passive ion redistribution alone. This addresses the downstream segment of the chain (transporter expression → Na^+^ exclusion). Second, phosphorylation-state analysis of the plasma-membrane H^+^-ATPase, specifically detection of the activating phosphorylation at the penultimate C-terminal threonine residue, would establish whether the ATP surplus measured under improved aeration is functionally coupled to proton-pump activation. This addresses the central node of the chain (ATP availability → H^+^-ATPase activity → proton-motive force), which is the critical link between the energy status documented in this study and the downstream ion transport processes. Third, ^22^Na^+^ influx/efflux kinetics measured in excised root segments under contrasting aeration treatments would provide direct, quantitative evidence of net Na^+^ exclusion rates at the root surface, allowing the strong correlation reported here (r = 0.83) to be interpreted in terms of actual transporter flux capacity rather than steady-state tissue ion accumulation. This addresses the functional output of the entire chain and would provide the most definitive test of whether aeration-driven ATP enhancement translates into measurable changes in Na^+^ transport.

One observation warrants particular attention: RA increased ATP by 18% but the K^+^/Na^+^ ratio by only 19%. Spatially heterogeneous oxygen supply (CV = 12.5%) may not sustain the continuous ATP production required for steady-state ion transport. Roots experiencing intermittent hypoxia may allocate available energy toward survival responses—aerenchyma formation, ethylene signaling cascades—rather than maintaining optimal ion homeostasis [5]. This could explain why RA underperformed despite measurable ATP gains.

### 4.3. Mesophyll Limitation and Photosynthetic Response

The combination of higher net photosynthesis (+23%) with lower intercellular CO_2_ (−15%) indicates that assimilation was limited by mesophyll conductance rather than by stomatal aperture. If stomata alone drove the photosynthesis increase, intercellular CO_2_ would have risen rather than fallen. The 29% increase in mesophyll conductance and its tighter correlation with net photosynthesis (r = 0.82) than with stomatal conductance (r = 0.58) support this interpretation [28,32].

The correlation between the K^+^/Na^+^ ratio and mesophyll conductance (r = 0.76) suggests that ionic balance may support mesophyll function through multiple pathways. Elevated cytoplasmic Na^+^ can shrink mesophyll cells, reduce aquaporin activity, and physically displace chloroplasts from optimal light-harvesting positions—all of which impair CO_2_ diffusion from substomatal cavities to chloroplast stroma [33]. Restoring ion homeostasis likely contributed to the observed photosynthetic improvements, though again we must acknowledge that correlation does not prove mechanism.

### 4.4. Practical Guidelines for System Design

These findings point toward a straightforward design principle: establish drainage first, then add oxygenation if desired. A single pipe network can serve both functions—drainage during high water-table periods, oxygenation once air-filled porosity has recovered above threshold. Several operational guidelines emerge from our results.

First, determine the site-specific air-filled porosity threshold before system installation. For sandy loam textures similar to ours, a target of approximately 10% provides a reasonable starting estimate; heavier clay soils may require 12–14%. Site-specific thresholds can be established by measuring relative gas diffusivity (Dp/D0) on undisturbed soil cores equilibrated to field moisture across a range of air-filled porosities, then fitting a segmented regression to identify the breakpoint in the Dp/D0–εa relationship. This single measurement campaign—requiring no more than 20–30 cores—can prevent the costly scenario of installing oxygenation infrastructure into soil that is not yet physically capable of supporting diffusion-based gas transport.

Second, design drainage spacing to achieve threshold air-filled porosity within 48 h of irrigation or significant rainfall. The Hooghoudt equation with measured saturated hydraulic conductivity provides a reasonable starting point for lateral spacing calculations, though site-specific adjustments may be needed.

Third, initiate oxygenation only after air-filled porosity has exceeded the threshold. Earlier injection largely wastes energy through preferential flow pathways. In our system, 48 h post-irrigation proved optimal, for three reasons: the percolation threshold—not the absolute porosity value—governs the transport regime, so the large gains in gas-phase connectivity arise at threshold crossing and increments above it yield diminishing returns; the cumulative duration of root hypoxia carries a real physiological cost, as delaying injection from 48 to 72 h extends sub-optimal oxygen exposure by approximately 24 h per irrigation cycle; and the agronomically relevant metric is the time-integrated dissolved oxygen across the full inter-irrigation interval, not the instantaneous concentration at the moment of injection—earlier initiation shifts the entire elevated-DO window forward, outweighing the marginal gain from injecting into slightly higher-porosity soil. Practitioners are advised to initiate oxygenation as soon as the site-specific threshold is confirmed to have been crossed, monitored by gravimetric sampling of undisturbed cores.

Fourth, monitor the drainage water iron concentration during the first few years of operation. If concentrations approach 5 mg L^−1^, schedule preventive pipe flushing to minimize ochre accumulation.

The economic case appears favorable: benefit–cost ratio exceeding 4, payback under 3 years, and robustness across sensitivity scenarios. Environmental co-benefits—32% reduction in global warming potential with no pollution trade-offs—provide additional justification where climate mitigation receives policy support.

### 4.5. Limitations and Future Directions

Several limitations of this study should be acknowledged. First, the air-filled porosity threshold of 10.2% identified in this study is specific to the sandy loam soil at our experimental site. While this value is consistent with published ranges for similar textures, it should not be applied directly to soils of contrasting texture, organic matter content, or structural condition without independent validation through site-specific Dp/D0–εa measurements.

Second, two growing seasons cannot address long-term system durability concerns. Our post-experiment inspection revealed minimal ochre accumulation, but monitoring over 10–15 years would provide more definitive evidence of system longevity.

Third, we tested one wheat cultivar at one location. Multi-site, multi-crop trials would improve confidence in the generalizability of our findings. Different crops with different rooting patterns and oxygen demands may show varying responses to drainage-oxygenation combinations.

Fourth, while drainage water nitrate concentrations met regulatory standards, there may be scope for further optimization through controlled-release fertilizers, nitrification inhibitors, or precision nitrogen management guided by crop sensing. These refinements could potentially reduce both environmental losses and input costs.

Fifth, the 48 h post-irrigation oxygenation initiation time was operationally defined based on threshold-crossing dynamics rather than empirically optimized. A formal timing trial—comparing initiation at, for example, 36, 48, 60, and 72 h post-irrigation—would quantify the trade-off between cumulative hypoxia duration and the marginal gains from injecting into progressively higher-porosity soil, and could refine the operational guideline offered in Section 4.4.

Finally, we focused on agronomic and physiological outcomes. A more complete sustainability assessment would include labor requirements, energy inputs over the full system lifecycle, and social acceptability among farming communities.

## 5. Conclusions

This field experiment provides evidence that subsurface drainage functions as a physical prerequisite for effective root-zone oxygenation in shallow-groundwater saline soils. Drainage raised air-filled porosity above the percolation threshold of 10.2% (95% CI: 9.1–11.3%), enabling formation of continuous gas-phase networks. Without drainage, oxygenation achieved only 4.2 mg L^−1^ dissolved oxygen with high spatial variability (CV 12.5%) and produced 7.2% yield increase. With drainage, oxygenation reached 6.8 mg L^−1^ (CV 6.8%) and increased yield by 30.7%. The synergy index of 1.40 indicates super-additive effects exceeding what simple addition of individual treatment benefits would predict.

Root ATP content increased 89% in the combined treatment, correlating strongly with 56% lower Na^+^ and 185% higher K^+^/Na^+^ ratio (r = 0.83). While these patterns are consistent with ATP-dependent ion exclusion mechanisms, molecular validation of transporter activity remains for future investigation.

Methane emissions declined 62%, and net global warming potential decreased 32% without pollution swapping. The system achieved a 2.9-year payback period and benefit–cost ratio of 4.08 under base-case assumptions, remaining economically viable across sensitivity scenarios.

For practitioners managing waterlogged saline soils, the practical message is clear: oxygenation systems implemented without prior drainage may disappoint. Restoring gas-phase connectivity through drainage infrastructure is the essential first step that enables subsequent oxygenation to function effectively.

## Figures and Tables

**Table 1:** Baseline soil properties (0–60 cm depth profile).

Depth (cm)	Bulk Density (g cm^−3^)	Sand (%)	Silt (%)	Clay (%)	EC_1:5_ (dS m^−1^)	ECe (dS m^−1^)	pH	Organic Matter (g kg^−1^)	ESP (%)	CEC (Cmolc kg^−1^)
0–20	1.42	58.3	28.5	13.2	1.20	6.0	8.4	11.5	12.5	14.2
20–40	1.48	55.8	30.2	14.0	1.32	6.6	8.5	9.8	13.8	13.8
40–60	1.52	52.5	32.1	15.4	1.45	7.3	8.6	7.5	15.2	13.5

Note: ECe values derived using site-specific conversion factor 5.0 (±0.35 SE). ESP, exchangeable sodium percentage; CEC, cation exchange capacity.

**Table 2:** Crop management schedule and field operations.

Operation	Timing	Specific Details
Sowing	Early October	cv. Jimai 22; 225 kg ha^−1^; 20 cm row spacing; 6 cm depth
Basal fertilization	Pre-sowing	63.6 kg N ha^−1^, 90 kg P_2_O_5_ ha^−1^, 75 kg K_2_O ha^−1^
Topdressing	Late March (joining)	95.4 kg N ha^−1^ as urea
Irrigation	Four key stages	45 mm per event: overwintering, jointing, anthesis, grain-filling; Total 180 mm
Oxygenation events	48 h post-irrigation	4 h duration; 30 L min^−1^ flow rate; ~80 m^3^ ha^−1^ per event; ~18 events per season
Harvest	Early June	Manual harvest from 3 × 1-m^2^ quadrats per plot

**Table 3:** Air-filled porosity dynamics at 10–20 cm depth following irrigation.

Treatment	24 h Post-Irrigation	48 h Post-Irrigation	72 h Post-Irrigation	96 h Post-Irrigation
CK	4.2 ± 0.3 b	5.8 ± 0.4 b	6.5 ± 0.5 b	7.2 ± 0.4 b
SD	8.5 ± 0.5 a	12.3 ± 0.6 a	14.2 ± 0.5 a	15.5 ± 0.4 a
RA	4.5 ± 0.4 b	6.2 ± 0.5 b	7.0 ± 0.4 b	7.8 ± 0.5 b
SD + RA	9.2 ± 0.6 a	13.5 ± 0.7 a	14.8 ± 0.6 a	15.8 ± 0.5 a

Values are mean ± SD (*n* = 9; three undisturbed cores per plot × three blocks). Different letters indicate significant differences (*p* < 0.05, Tukey’s HSD).

**Table 4:** Relative gas diffusivity and air-filled porosity relationship at 48 h post-irrigation.

Treatment	Dp/D0 (Dimensionless)	εa (%)	Ratio to 0.02 Threshold
CK	0.008 ± 0.002 b	5.8 ± 0.4	0.40
SD	0.042 ± 0.006 a	12.3 ± 0.6	2.10
RA	0.010 ± 0.003 b	6.2 ± 0.5	0.50
SD + RA	0.048 ± 0.007 a	13.5 ± 0.7	2.40

Dp/D0: relative gas diffusivity. Values are mean ± SD (*n* = 9). Different letters indicate significant differences (*p* < 0.05).

**Table 5:** Changes in root-zone soil salinity over a two-year experimental period.

Treatment	Baseline EC_1:5_ (dS m^−1^)	Final EC_1:5_ (dS m^−1^)	Absolute Change (dS m^−1^)	Relative Change (%)
CK	1.20 ± 0.08	1.17 ± 0.09	−0.03	−2.5
SD	1.18 ± 0.07	0.98 ± 0.08	−0.20	−16.9
RA	1.21 ± 0.08	1.17 ± 0.09	−0.04	−3.3
SD + RA	1.19 ± 0.07	0.93 ± 0.08	−0.26	−21.8

Measurements at 0–20 cm depth. Values represent mean ± SD of 15 sampling points per treatment.

**Table 6:** Comprehensive physiological, photosynthetic, and yield parameters at anthesis.

Parameter	Unit	CK	SD	RA	SD + RA
Oxygen environment					
DO (peak, T3)	mg L^−1^	2.6 ± 0.3 c	4.0 ± 0.3 b	4.2 ± 0.4 b	6.8 ± 0.4 a
DO (season mean)	mg L^−1^	2.6 c	3.8 b	3.4 b	5.6 a
Eh	mV	+148 ± 15 c	+178 ± 12 b	+162 ± 14 bc	+205 ± 11 a
Root energy and ions					
Root ATP	μmol g^−1^ FW	1.56 ± 0.12 d	2.22 ± 0.15 b	1.84 ± 0.14 c	2.95 ± 0.18 a
Root Na^+^	mg g^−1^ DW	18.5 ± 1.2 a	12.8 ± 0.9 b	16.2 ± 1.1 a	8.2 ± 0.7 c
Root K^+^	mg g^−1^ DW	22.5 ± 1.5 c	25.8 ± 1.4 b	23.2 ± 1.3 c	28.5 ± 1.6 a
K^+^/Na^+^ ratio	—	1.22 ± 0.08 d	2.02 ± 0.12 b	1.43 ± 0.10 c	3.48 ± 0.18 a
Photosynthesis					
Pn	μmol m^−2^ s^−1^	17.3 ± 0.8 c	18.8 ± 0.7 b	17.9 ± 0.9 bc	21.2 ± 0.8 a
Gs	mol m^−2^ s^−1^	0.285 ± 0.020 c	0.342 ± 0.022 b	0.305 ± 0.021 c	0.400 ± 0.028 a
Gm	mol m^−2^ s^−1^	0.144 ± 0.012 c	0.162 ± 0.013 b	0.152 ± 0.012 bc	0.185 ± 0.014 a
Ci	μmol mol^−1^	268 ± 12 a	245 ± 10 b	258 ± 11 ab	229 ± 9 c
Root development					
Root length density	cm cm^−3^	2.19 ± 0.15 c	2.85 ± 0.18 b	2.38 ± 0.16 c	3.25 ± 0.20 a
Root dry weight	g plant^−1^	1.10 ± 0.08 d	1.42 ± 0.10 b	1.23 ± 0.09 c	1.78 ± 0.12 a
Yield					
Grain yield	kg ha^−1^	5798 ± 285 d	6652 ± 310 b	6215 ± 295 c	7580 ± 325 a
Water productivity	kg m^−3^	1.38 ± 0.08 c	1.52 ± 0.09 b	1.45 ± 0.08 bc	1.74 ± 0.10 a
PFPN	kg kg^−1^	36.5 ± 2.1 c	41.8 ± 2.3 b	39.1 ± 2.2 bc	47.7 ± 2.5 a
Spike number	10^4^ ha^−1^	422 ± 18 c	448 ± 20 b	432 ± 19 c	465 ± 22 a
Grains per spike	grains/spikes	33.8 ± 1.5 c	35.8 ± 1.6 b	34.5 ± 1.4 c	37.8 ± 1.8 a
Thousand-grain wt	g	41.2 ± 1.8 c	42.8 ± 1.9 b	41.8 ± 1.7 bc	44.5 ± 2.0 a

Values are mean ± SD (*n* = 9). Different letters indicate significant differences (*p* < 0.05). FW, fresh weight; DW, dry weight; Pn, net photosynthesis; Gs, stomatal conductance; Gm, mesophyll conductance; Ci, intercellular CO_2_; PFPN, partial factor productivity of nitrogen.

**Table 7:** Drainage water quality characteristics.

Parameter	Year 1	Year 2	Mean ± SD
NO_3_^−^-N concentration (mg L^−1^)			
Season mean	8.5 ± 1.2	9.2 ± 1.4	8.9 ± 1.3
Peak (post-fertilization)	12.3 ± 2.1	14.8 ± 2.5	13.6 ± 2.3
Minimum	4.2 ± 0.8	4.8 ± 0.9	4.5 ± 0.9
Drainage volume (mm season^−1^)	42 ± 5	48 ± 6	45 ± 6
Nitrogen load (kg N ha^−1^)	4.8 ± 0.6	5.2 ± 0.7	5.0 ± 0.7
Water quality compliance			
China Class III (20 mg L^−1^)	100%	100%	100%
WHO guideline (50 mg L^−1^)	100%	100%	100%

Values represent mean ± SD of samples collected after each irrigation event (*n* = 4 events × 3 replicates × 2 years = 24 per parameter).

**Table 8:** Two-way ANOVA results for key response variables.

Response Variable	Drainage (D)	Aeration (A)	D × A Interaction
Dissolved oxygen (T3)	85.2 ***	42.6 ***	25.6 ***
K^+^/Na^+^ ratio	68.5 ***	35.2 ***	22.4 ***
Root ATP content	52.8 ***	28.4 ***	18.5 ***
Grain yield	48.2 ***	22.6 ***	16.8 ***
Methane emission	38.5 ***	18.2 ***	14.2 ***
Root length density	44.6 ***	20.8 ***	12.5 **
Net photosynthesis	36.2 ***	16.5 ***	11.8 **

F-values from two-way ANOVA with block as a random effect. *** *p* < 0.001; ** *p* < 0.01.

**Table 9:** Economic analysis of an integrated drainage-oxygenation system.

Economic Parameter	Value	Unit
Capital costs		
Drainage pipes and fittings	4200	CNY ha^−1^
Oxygenation equipment	3600	CNY ha^−1^
Installation labor	1000	CNY ha^−1^
Total initial investment	8800	CNY ha^−1^
Annual costs		
Operating cost	400	CNY ha^−1^ year^−1^
Depreciation (15-year)	587	CNY ha^−1^ year^−1^
Total annual cost	987	CNY ha^−1^ year^−1^
Financial metrics		
Simple payback period	2.2	years
Discounted payback (6% rate)	2.9	years
Net present value (15 years)	31,540	CNY ha^−1^
Benefit–cost ratio	4.08	—

CNY = Chinese Yuan. USD equivalents were calculated at the CNY/USD exchange rate of ~7.2, as published by the People’s Bank of China in June 2024, coinciding with the period of economic data collection. All primary economic metrics (BCR, NPV, payback period) are denominated in CNY and are therefore invariant to exchange rate fluctuation; USD figures are provided for indicative reference only.

## Data Availability

The data presented in this study are available on request from the corresponding author due to privacy.
